# Investigation of Volatile Components and Assessment of Antioxidant Potential in Seven Lamiaceae Plant Hydrosols

**DOI:** 10.3390/molecules29010145

**Published:** 2023-12-26

**Authors:** Ziwei Xin, Wenjia Wang, Weizong Yang, Yajie Li, Lixin Niu, Yanlong Zhang

**Affiliations:** College of Landscape Architecture and Arts, Northwest A&F University, Yangling 712100, China; xinziwei2020@nwafu.edu.cn (Z.X.); ww10617@126.com (W.W.); yangweizong@nwafu.edu.cn (W.Y.); liyajie@nwafu.edu.cn (Y.L.)

**Keywords:** Lamiaceae, hydrosol, GC-MS, volatile components, antioxidant activity

## Abstract

Aromatic plants of the family Lamiaceae, especially of the genus Thymus, have promising antioxidant applications in pharmacology, medicine, food, cosmetology, and aromatherapy. Hydrosols (HDs) were extracted by hydrodistillation from seven species of Lamiaceae, including *Thymus vulgaris*, *Thymus mongolicus*, *Mentha × piperita*, *Melissa officinalis*, *Rosmarinus officinali*, *Salvia elegans*, and *Leonurus artemisia*. In total, 369 volatile components were determined and analyzed by gas chromatography–mass spectrometry (GC-MS). Among them, alcohols (2.86–28.48%), ethers (2.46–10.69%), and phenols (0.11–21.78%) constituted a large proportion, mainly linalool (0.28–19.27%), eucalyptol (0.16–6.97%), thymol (0–19.54%), and carvacrol (0–26.82%). Multivariate statistical analyses were performed and 27 differential metabolites were screened. Three different methods (ABTS^+•^, DPPH^•^, and FRAP) were used to determine the in vitro antioxidant activity of seven HDs. *Thymus vulgaris* hydrosols (Tv HDs) and *Thymus mongolicus* hydrosols (Tm HDs) had the strongest antioxidant activity and their stronger antioxidant capacity was related to their high levels of phenolic constituents, mainly thymol. The antioxidant activity of the other five Lamiaceae HDs was associated with their high alcohol (mainly linalool and eucalyptol) content, and the alcohol constituents may synergistically affect their antioxidant capacity. Therefore, the present study suggests that Lamiaceae plants can be utilized as antioxidant products or antioxidants in different industrial sectors including pharmaceuticals, food, cosmetics, and agrochemicals.

## 1. Introduction

Secondary metabolites are valuable natural compounds of natural origin with important biological activities [1]. Hydrosols are saturated distillates obtained from the roots, stems, leaves, flowers, and other different parts of aromatic plants through steam distillation [2]. Hydrosols were initially believed to be byproducts obtained from the production of essential oils but they were later found to contain many active ingredients, such as monoterpenoids, aromatic hydrocarbons, polyphenols, ethers, and aldehydes [3]. Essential oils and hydrosols have antioxidant properties and using them as natural antioxidants is increasingly gaining attention [4,5,6]. Synthetic antioxidants [7], such as BHA (Butylated hydroxyanisole) and BHT (2,6-Di-tert-butyl-4-methylphenol), may be potentially harmful to human health [8]. At present, hydrosols are recognized as safe compounds, and their reasonable use does not adversely affect human health [9,10]. Moreover, their production costs are much lower than those of essential oils, and water-soluble components are more easily absorbed by the human body [11,12]. Therefore, the market share of hydrosol is constantly expanding, and the development of hydrosol products necessitates further research.

The Lamiaceae family (Lamiaceae) contains many valuable medicinal plants, and the essential oils and hydrosols of Lamiaceae are rich in secondary metabolites [13]. In addition, they are also useful in natural medicine, pharmaceuticals, cosmetics, and fragrances [14]. During these years, there has been a growing interest in plants of the Lamiaceae family and their potential applications as a source of nutritious foods and medicines [15]. Many species of Lamiaceae plants are known and their different parts such as flowers, stems, and leaves contain various volatile substances such as aromatic alcohols, terpenoids, and phenols [16]. Previous studies have found that aromatic plants in the Lamiaceae family contain polyphenols, diterpenoids, phenolic acids, flavonoids, aromatic alcohols, volatile terpenoids, and other substances [17]. Various hydrosols of the Thymus genus have insecticidal properties, which can be used as an alternative to synthetic chemical insecticides [18]. Therefore, Lamiaceae plants and their extracts can be used as potential additives in the field of food, flavorings, cosmetics, and pharmaceuticals, possessing a broad economic value.

Although essential oils, as well as antioxidants extracted from Lamiaceae plants, are useful in natural medicine, pharmaceuticals, cosmetics, and fragrances [19,20,21,22], research on hydrosols and the physiological activity determination of various aromatic plants in the Lamiaceae family is scarce. In this study, we extracted hydrosols from seven aromatic plants in the Lamiaceae family including *Thymus vulgaris*, *Thymus mongolicus*, *Mentha × piperita*, *Melissa officinalis*, *Rosmarinus officinali*, *Salvia elegans*, and *Leonurus artemisia*. Furthermore, the volatile components of these seven hydrosols were detected and identified, and the antioxidant capacity of seven Lamiaceae hydrosols to synthesize the DPPH and ABTS free radicals, as well as their ability to reduce ferric ions (FRAP), were also demonstrated.

## 2. Results and Discussion

### 2.1. GC-MS Analysis of Volatile Components of Seven Lamiaceae Hydrosols

**Figure 1:**
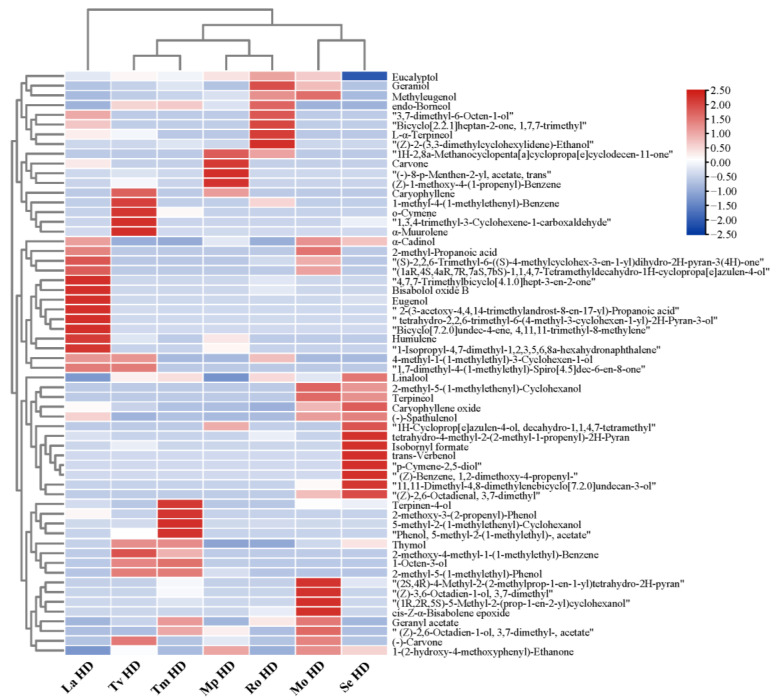
Relative content (%) of main volatile compounds from hydrosols of seven Lamiaceae plants.

The main volatile components identified from seven kinds of hydrosols extracted from *Thymus vulgaris* (Tv HD), *Thymus mongolicus* (Tm HD), *Mentha × piperita* (Mp HD), *Melissa officinalis* (Mo HD), *Rosmarinus officinali* (Ro HD), *Salvia elegans* (Se HD), and *Leonurus artemisia* (La HD) are shown in Figure 1 and Supplementary Table S1, and the total ion flow diagrams of the seven hydrosols are shown in Supplementary Figure S1. A total of 369 volatile components were identified in the seven Lamiaceae hydrosols by GC-MS analysis, among which Tm HDs contained the largest number of volatile components, with 113 types, and Mo HDs contained the smallest number of volatiles, at 47. The identified volatile components included various types of substances such as esters, olefins, alkanes, ethers, aromatic hydrocarbons, ketones, phenols, alcohols, etc., and there were obvious differences in the contents of different substances (Table 1). Among them, alcohols (2.86–28.48%), ethers (2.46–10.69%), and phenols (0.11–21.78%) accounted for a relatively large proportion of the substances, mainly comprising linalool (0.28–19.27%), (0.28–19.27%), eucalyptol (0.16–6.97%), thymol (0–19.54%), and carvacrol (0–26.82%), while aldehydes were less predominant. (-)-Eudesmol, stigmasterol oxide, eucalyptol, and linalool were the common volatile constituents in the seven hydrosols, and the relative contents of these substances varied among different hydrosols. Among them, the volatile components of Tv HDs and Tm HDs had the highest content of phenolic substances, ketones had the highest content among Mp HDs, Mo HDs, and Ro HDs, and alcohols had the highest content among Se HDs and La HDs. Significant differences in the content of thymol, p-cymene, and terpinene in essential oils from different origins of Thymus mongolicus from Poland, Iran, Spain, and Italy, which can be attributed, to a large extent, to the different chemical types of the aromatic plant species, were reported by Boruga et al. [23]. This is in line with the findings of the present study that the types of volatile compounds differed among the different species of aromatic plants of the family Lamiaceae.

### 2.2. Multivariate Analysis of Volatile Constituents of Seven Hydrosols from the Lamiaceae Family

**Figure 2:**
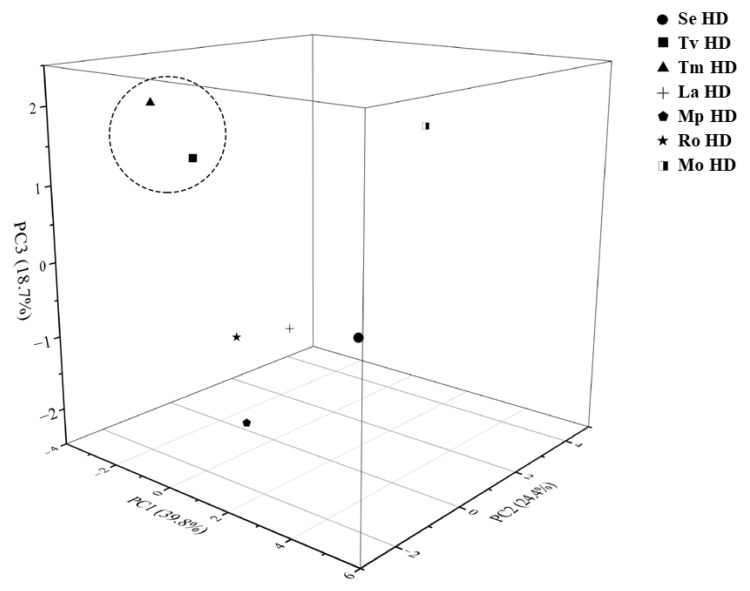
Three-dimensional score plot of principal components of seven Lamiaceae plant hydrosols.

**Figure 3:**
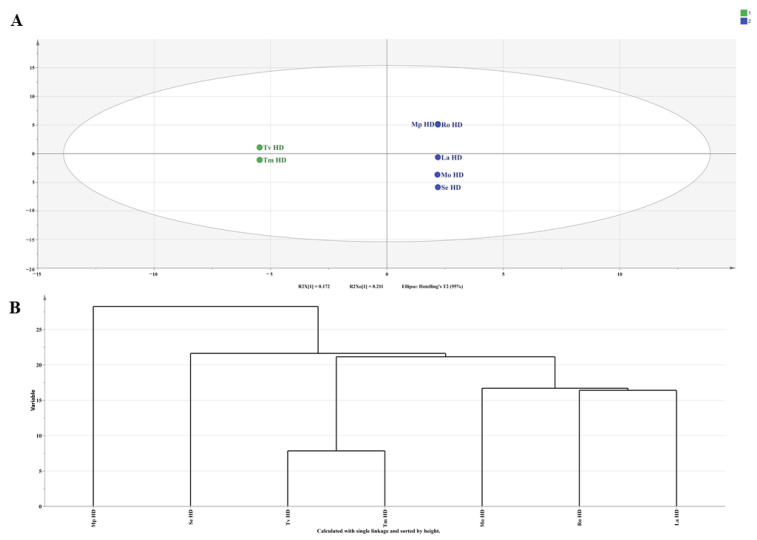
Multivariate statistical analysis of volatile components from seven Lamiaceae plant hydrosols: (**A**) OPLS-DA score scatter plot; (**B**) HCA plot for principal components PCA.

**Figure 4:**
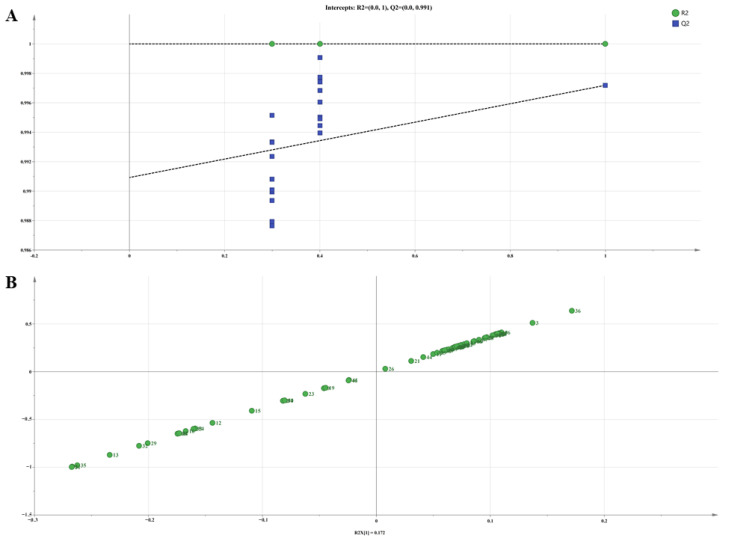
(**A**) Plot for permutation test; (**B**) s-plot for OPLS-DA.

To further assess the volatile constituent profiles of the seven Lamiaceae hydrosols, we employed principal component analysis (PCA), cluster analysis (HCA), and orthogonal partial least squares discriminant analysis (OPLS-DA) to conduct multivariate statistical analyses. PCA is one of the most applied data dimensionality reduction analysis methods. In PCA, the eigenvalues of PC1, PC2, and PC3 were all greater than 1, and the cumulative variance contribution ratio was 82.929%, indicating significant biochemical variations among the samples. As shown in Figure 2, the variance contribution rate of PC1 was 39.800%, which mainly integrated the content of (-)-eucalyptol and stigmasterol oxide, indicating that these two substances can be used as the main compounds for identifying the volatile components in the seven hydrosols; the variance contribution rate of PC2 and PC3 were 24.398% and 18.731%, respectively. Tv HDs and Tm HDs were clustered together, indicating that the types and contents of the main components of the two hydrosols were similar, which may be because both plants belong to the Thymus. There was no significant difference between the two hydrosols of the genus Thymus and other hydrosols in the PC1 dimension, while there was a significant difference in the PC3 dimension, which may be because the main component of PC3 was thymol, and the contents of thymol in Tv HDs and Tm HDs were 18.84% and 19.54%, respectively, which were significantly higher than that of the thymol in the hydrosols of the other five species of the family Lamiaceae (0–5.32%). As shown in Figure 3A, we performed OPLS-DA analysis using SIMCA 14.1, and the Tv HDs and Tm HDs were distributed on both sides of the 95% confidence interval with the other five Lamiaceae HDs. This is consistent with the results of PCA, and HCA (Figure 3B) supports this classification. The results of R2, Q2, and s-plot are shown in Figure 4. Further, we screened for differential metabolites by t-test at variable importance scores (VIP) > 1 and *p* < 0.05 to elucidate specific differences in volatile constituents among the seven Lamiaceae hydrosols. Twenty-seven differential metabolites were screened (Supplementary Table S2), and ten volatile compounds with a relative content of more than 1% were L-hydroquinone, stigmasterol oxide, thymol, α-bisacodyl alcohol, terpinen-4-ol, 1-octen-3-ol, 2-methoxy-4-methyl-1-(1-methylethyl)-benzene, cyclohexanol, and 2-methyl-5-isopropylphenol.

### 2.3. In Vitro Antioxidant Activity Analysis of Seven Lamiaceae Hydrosols

**Figure 5:**
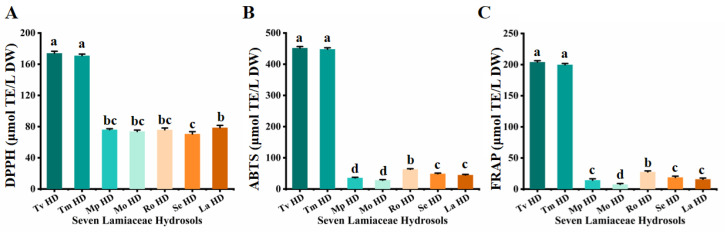
In vitro antioxidant activities of seven Lamiaceae plant hydrosols. (**A**) ABTS^+•^ assays. (**B**) DPPH^•^ assays. (**C**) FRAP assays. Column and error bar represent mean values and standard deviation (*n* = 3), respectively. Different letters in each column (a–d) denote significant inter-species differences (*p* < 0.05). Different colors in each column indicate different Lamiaceae species.

To more accurately assess the antioxidant capacity of the seven Lamiaceae hydrosols, three different methods (DPPH^•^, ABTS^+•^, and FRAP) were used to determine the in vitro antioxidant activity of the hydrosols (Table 2). The results of the DPPH^•^ assay showed that the antioxidant activities of Tv HDs and Tm HDs were relatively close to each other, reaching 174.04 μmol TE/L DW and 170.92 μmol TE/L DW, which were the best antioxidant properties among the seven hydrosols. The other five Lamiaceae hydrosols also showed definite antioxidant activities, with La HDs, Ro HDs and Mp HDs showing slightly stronger activities (Figure 5A). The results of the ABTS^+•^ assay were in agreement with those of DPPH^•^, and both Tv HDs and Tm HDs showed the best antioxidant properties, reaching 451.79 μmol/L and 447.93 μmol TE/L DW, respectively, followed by Ro HDs, reaching 63.06 μmol TE/L DW, whereas the worst antioxidant activity was found in Mo HDs, which had 28.00 μmol TE/L DW (Figure 5B). Further, the results of the FRAP assay similarly supported this conclusion. Tv HDs and Tm HDs showed the best antioxidant activity of 204.02 μmol TE/L DW and 199.75 μmol TE/L DW, while Mo HDs showed the worst activity of 7.52 μmol TE/L DW (Figure 5C). Based on the above results, we concluded that Thymus vulgaris and Thymus mongolicus can be used as the preferred species for the development of Lamiaceae aromatic plant hydrosols as antioxidant products or antioxidants. The development of hydrosols from the genus Thymus should be studied further.

Gil et al. [24] found that the values of the ABTS test were usually significantly higher than those of the DPPH test, and Aebisher et al. [8] showed that the scavenging activity of the essential oils of the family Lamiaceae was significantly higher for ABTS radicals than for DPPH. This is consistent with the results of the ABTS and DPPH performances of Tv HDs and Tm HDs in the present study, where the scavenging of ABTS free radicals by the two Thymus spp. hydrosols were almost up to twice the scavenging rates of DPPHS free radicals. The Lamiaceae family is universally considered as an important source of natural antioxidants [25]. Back in the 1950s, Chipault and co-workers (1952) [26] first identified the ability of rosemary and sage to inhibit deleterious oxidative reactions. Thymus pulegioides L. essential oil, which was grown in Western Romania, was also reported to have good antioxidant properties, and the compound that contributed most to its antioxidant efficacy was also thymol (22.89%) [27]. Matilda Rădulescu et al. identified 36 compounds in the Melissa officinalis subsp. officinalis essential oil and verified their antioxidant activity by ABTS and DPPH assays. Moreover, Melissa officinalis and Pastianica sylvestris essential oil were also reported to exhibit a strong inhibitory effect in the β-carotene bleaching assay, even compared to butylated hydroxyanisole (BHA) and butylated hydroxytoluene (BHT) [28,29]. Moreover, the aerial material of most aromatic plants belonging to the Lamiaceae family distributed in different regions of the globe, such as members of *Salvia viridis* [30], *Thymus pubescens* [30], *Mentha × smithiana* [31], *Thymbra spicata* [32], and *Ziziphora clinopodioid* [33], etc., are added to foods for their antioxidant properties and are often consumed as herbal teas, or added to cosmetics as a natural antioxidant.

### 2.4. Correlation Analysis between Volatile Components and Antioxidant Activity of Seven Lamiaceae Hydrosols

**Figure 6:**
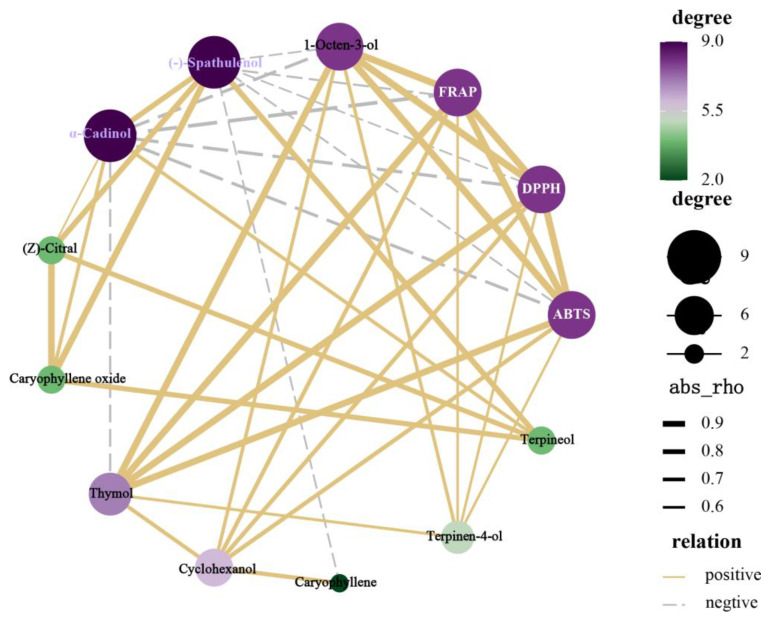
Correlation network between bioactive compounds and antioxidant activities in seven Lamiaceae plant hydrosols.

Hydrosols exhibit biological activities corresponding to the presence of active ingredients [34]. Therefore, as shown in Figure 6, we used Pearson’s correlation analysis to further reveal the potential relationship between the differential volatile metabolites and antioxidant activity of the seven Lamiaceae hydrosols. In this study, we considered that the active constituents that had a greater influence on the antioxidant activity of the Lamiaceae hydrosols were phenolic and alcoholic constituents such as thymol, terpinene-4-ol, 1-octen-3-ol, and cyclohexanol, which were detected at the threshold level. The thickness of the line in the correlation network represents the strength of the correlation, and we found that thymol and 1-octen-3-ol had the strongest correlation with the antioxidant activity of Lamiaceae hydrosols. We speculate that the stronger antioxidant capacity of Tv HDs and Tm HDs is related to their high levels of phenolic constituents. Phenolic compounds have a greater impact on antioxidant capacity [35,36]. This is in line with previous studies on Thymus vulgaris L. hydrolate extracts, which showed that Thymus vulgaris L. hydrosol had a higher content of thymol, and a correspondingly high antioxidant capacity [37]. Linalool and eucalyptol, which are common to all seven Lamiaceae hydrosols in this study, also showed a strong correlation with antioxidant activity, indicating that linalool- and eucalyptol-based alcohols also contribute to the antioxidant activity of purees. Linalool, as an important aromatic compound, has a higher percentage of hydrosols [38], implying that it can be completely dissolved in water and may exhibit better antioxidant activity [39]. Eucalyptol, although the main component in essential oils, is present in hydrosols, and its solubility in water at 25 °C is nearly 3.5 × 103 mg/mL [40]. Eucalyptol can scavenge oxygen radicals and eliminate hydroxyl radicals, making it a highly effective antioxidant [41]. Therefore, the antioxidant capacity of the other Lamiaceae hydrosols may be due to their higher levels of alcohol constituents. Although the other active ingredients did not show a direct and strong correlation with antioxidant activity, they may act synergistically with phenolic and alcoholic components to influence the antioxidant capacity of the hydrosols. Therefore, the present study suggests that aromatic plants of the family Lamiaceae, especially those of the genus Thymus, especially Thymus vulgaris and Thymus mongolicus, have good prospects as antioxidants. Although the present study demonstrated the potential application of Lamiaceae hydrosol as an antioxidant, we should also carry out in vivo and in vitro validation of the antioxidant activity of Lamiaceae extracts in future practical applications to ensure the safety of its application. This research will be enhanced by further studies in the future.

## 3. Materials and Methods

### 3.1. Plant Materials

All seven Lamiaceae plant materials were harvested from the “Yuningxiang” planting base in Heyang County, Weinan City, Shaanxi Province, including *Thymus vulgaris*, *Thymus mongolicus*, *Mentha × piperita*, *Melissa officinalis*, *Rosmarinus officinali*, *Salvia elegans*, and *Leonurus artemisia*. Lamiaceae plants with vigorous growth of branches and foliage and strong green foliage color were selected to be harvested before the full bloom period in May and June (Table 3).

### 3.2. Sample Extraction

Branches and leaves of seven Lamiaceae species were dried using a vacuum freeze dryer (LGJ-10D, Sihuan Scientific Instrument Factory, Beijing, China). The lyophilized samples were ground to a fine powder with a high-speed disintegrator (Model SF-2000, Chinese Traditional Medicine Machine Works, Shanghai, China).

For extracting hydrosol, the method described for peony hydrosol extraction was followed [42], which was optimized by our group. Seven Lamiaceae species plants’ hydrosols were prepared by steam distillation. In summary, a 2.5 L round-bottom flask was filled with roughly 200 g of lyophilized materials. Using a heating mantel, 1.2 L of distilled water was added to the flask and brought to a boil. Following 4 h of distillation, condensation, and reflux, the separator gathered the liquid hydrosols. Until additional analysis, the hydrosols were maintained at 4 °C and airtight-sealed with the parafilms.

### 3.3. Chemicals

The 3-octanol, Water-soluble vitamin E (Trolox), 2,2′-azino-bis(3-ethylbenzothiazoline-6-sulfonic acid (ABTS), 1,1-diphenyl-2-picrylhydrazyl radical (DPPH), and 2,4,6-Tri(2-pyridyl)-s-triazine (TPTZ) were bought from Shanghai Yuanye Bio-technology Co., Ltd. (Shanghai, China). Fluorescein was obtained from Chengdu Kelong Co., Ltd. (Chengdu, China). Chromatography-grade methanol and n-hexane were purchased from Tianjin Bodi Chemical Co., Ltd. (Tianjin, China). The other reagents used in this study were of analytical grade and purchased from Xi’an Sanpu Chemical Reagent Co., Ltd. (Xi’an, China) [43].

### 3.4. GC-MS Analysis

GC-MS analysis was improved by referring to the method of Quan et al. [44]. We used a TRACE 1310ISQLT gas chromatograph–mass spectrometer equipped with a Thermo TG-5MS (30 m × 0.25 mm × 0.25 μm) capillary column for the experiments.

Chromatographic conditions: In the heating program, the starting temperature was 40 °C, with a hold for 3 min. The temperature was increased to 210 °C at a rate of 3.0 °C/min, and held for 2 min. The temperature was increased to 290 °C at a rate of 20 °C/min, held for 15 min, and the operation was performed for 80.75 min. In the constant-flow mode, the carrier gas was high-purity helium (purity > 99.999%) with an inlet volume of 1 μL.

MS conditions were as follows: electron bombardment ion (EI) source, electron energy of 70 eV, transmission line temperature of 230 °C, quadrupole mass analyzer, positive ion scanning, and mass scanning range of 45–600 amu.

The total ion chromatograms of the volatile components were obtained by GC-MS, and the volatile components were characterized by ONLINE search, referring to the NIST2014 standard spectra, and combining them with the relative retention time. The relevant literature and data were reviewed and the relative contents of the volatile components were analyzed by the peak-area normalization method [45,46].

### 3.5. Assessment of Antioxidant Activity with ABTS^+•^, DPPH^•^, and FRAP Assays

#### 3.5.1. Determination of DPPH Free Radical Scavenging Capacity of Seven Lamiaceae Plant Hydrosols

The scavenging activity of DPPH radicals was assessed by referring to the method described by Dudonné et al. with slight modifications [20]. Briefly, 2 mL of hydrosol was added to 0.95 mL of DPPH solution (2.0 × 10^−4^ mol/L). The reaction was carried out at 37 °C and protected in the dark for 30 min. Specifically, 200 μL of the reaction solution was added to a 96-well ELISA plate, and the absorbance value was measured at 517 nm.

#### 3.5.2. Determination of ABTS Radical Scavenging Ability of Seven Lamiaceae Plant Hydrosols

We referred to the ABTS^+•^ determination method described by Re et al. [47] with slight modifications. The ABTS solution was obtained by mixing 7.3 mM ABTS and 4 mM K_2_S_2_O_8_, and the reaction was implemented at 25 °C in the dark for 15 h. The solution was diluted to obtain an ABTS^+•^ solution with an absorbance value of 0.7 at a wavelength of 734 nm. The diluted ABTS^+•^ solution and 200 μL of the hydrosol sample were mixed well, the reaction was protected from light for 8 min, and the absorbance value was measured at 734 nm.

#### 3.5.3. Determination of Ferric-Reducing Antioxidant Power of Seven Lamiaceae Plant Hydrosols

The ferric-reducing antioxidant power (FRAP) assay of seven Lamiaceae hydrosols was performed following the method proposed by Ou et al. with slight modifications [48]. The FRAP solution was obtained by combining 2.5 mL of 10 mM TPTZ solution in 40 mM HCl, 2.5 mL of 20 mM ferric chloride, and 25 mL of 0.3 M acetate buffer, and heated to a constant temperature of 37 °C in a water bath. The FRAP solution and 200 μL of the hydrosol sample were mixed well, the reaction was protected from light for 20 min, and the absorbance value was measured at 593 nm.

Calibration curves were established using Trolox as the standard for all above assays, and the results were expressed as l μmol of TE per 1 L of the sample (μmol TE/L DW).

### 3.6. Statistical Analysis

All experiments were performed in three replicates, and values are expressed as mean ± standard deviation (SD). Analyses for statistical significance were performed using SPSS 21.0. One-way analysis of variance (ANOVA) and Duncan’s multiple extreme variance test were used for statistical treatment (*p* < 0.05) [49]. HCA analysis was performed using standardized variables with Ward chain and squared Euclidean distance, and the correlation matrix was subjected to PCA and OPLS-DA. PCA, HCA, and OPLS-DA models were generated utilizing SIMCA 14.1. Correlation analysis was generated utilizing the OmicStudio tool [46,50].

## 4. Conclusions

In this study, we determined the volatile components and antioxidant activities of seven Lamiaceae hydrosols. A total of 369 volatile components were identified and analyzed by GC-MS, among which alcohols (2.86–28.48%), ethers (2.46–10.69%), and phenols (0.11–21.78%) accounted for a relatively large proportion of the substances, mainly comprising linalool (0.28–19.27%), (0.28–19.27%), eucalyptol (0.16–6.97%), thymol (0–19.54%), and carvacrol (0–26.82%). (-)-Eudesmol, stigmasterol oxide, eucalyptol, and linalool were the common volatile constituents in the seven hydrosols. The high content of phenolic compounds and alcohols in Lamiaceae hydrosols confers strong antioxidant activity. The results of antioxidant assays showed that the antioxidant activities of Tv HDs and Tm HDs were relatively close to each other, and were the best antioxidant performers among the seven hydrosols, while the other five Lamiaceae hydrosols also showed definite antioxidant activities, with La HDs, Ro HDs, and Mp HDs showing slightly stronger activities. The DPPH^•^ values of Tv HDs and Tm HDs, respectively, reached 174.04 μmol TE/L DW and 170.92 μmol TE/LDW, and ABTS^+•^ values reached 451.79 μmol TE/L and 447.93 μmol TE/LDW, respectively, in addition to FRAP results of 204.02 μmol TE/L DW and 199.75 μ mol TE/LDW. Thymol and 1-octen-3-ol had the strongest correlation with the antioxidant activity of Lamiaceae hydrosols, and we speculate that the stronger antioxidant capacities of Tv HDs and Tm HDs are related to their high levels of phenolic constituents. The antioxidant activity of the other five Lamiaceae HDs was associated with their high alcohol (mainly linalool and eucalyptol) content, and the alcohol constituents may synergistically affect their antioxidant capacity. Therefore, the present study suggests that Lamiaceae plants, especially of the genus Thymus, can be utilized as antioxidant products or antioxidants in different industrial sectors including pharmaceuticals, food, cosmetics, aromatherapy, and agrochemicals.

## Figures and Tables

**Table 1:** Relative content (%) of components belonging to different biochemical classes in the seven Lamiaceae hydrosols.

	Tv HD	Tm HD	Mp HD	Mo HD	Ro HD	Se HD	La HD
Esters	0.84	0.7	0.5	3.29	2.47	0.84	2.64
Olefins	0.34	0.02	0.71	0.71	0.18	0.22	4.97
Alkanes	tr	0.12	0.04	0.1	tr	0.03	0.31
Ethers	10.57	7.19	5.88	10.69	7.7	2.46	3.75
Aromatic Hydrocarbons	2.51	0.36	0.13	-	0.02	-	-
Ketones	2.21	2.61	39.62	7.75	18.46	11.72	16.67
Phenols	20.78	21.78	0.25	1.37	0.11	5.51	4.41
Alcohols	12.73	16.52	2.86	25.55	21.03	28.48	11.5
Others	0.01	0.26	0.01	0.01	0.01	0.56	2.31

Note: “-” indicates that the substance was not detected under analytical conditions used; “tr”, trace (<0.01%).

**Table 2:** Antioxidant properties of seven Lamiaceae hydrosols (*n* = 3 ± SD).

	DPPH (μmol TE/L DW)	ABTS (μmol TE/L DW)	FRAP (μmol TE/L DW)
Tv HD	174.04 ± 2.60 ^a^	451.79 ± 4.91 ^a^	204.02 ± 2.35 ^a^
Tm HD	170.92 ± 2.07 ^a^	447.93 ± 4.74 ^a^	199.75 ± 2.04 ^a^
Mp HD	76.00 ± 1.33 ^bc^	35.73 ± 1.86 ^d^	14.26 ± 2.47 ^c^
Mo HD	73.68 ± 1.91 ^bc^	28.00 ± 2.17 ^d^	7.52 ± 1.56 ^d^
Ro HD	75.84 ± 2.41 ^bc^	63.06 ± 2.05 ^b^	27.57 ± 1.72 ^b^
Se HD	70.53 ± 3.02 ^c^	48.89 ± 2.18 ^c^	18.72 ± 2.15 ^c^
La HD	78.59 ± 3.05 ^b^	44.86 ± 2.13 ^c^	15.81 ± 2.06 ^c^

Different superscript letters in each column indicate significant differences (*p* < 0.05).

**Table 3:** Information on the seven Lamiaceae plant hydrosols.

No.	Species	Abbreviations	Section	Extraction Parts
1	*Thymus vulgaris*	Tv HD	Lamiaceae	Branches and leaves
2	*Thymus mongolicus*	Tm HD		
3	*Mentha × piperita*	Mp HD		
4	*Melissa officinalis*	Mo HD		
5	*Rosmarinus officinali*	Ro HD		
6	*Salvia elegans*	Se HD		
7	*Leonurus artemisia*	La HD		

## Data Availability

All relevant data are included in the manuscript and its supporting materials further inquiries can be directed to the corresponding authors.

## Supplementary Materials

The following supporting information can be downloaded at: https://www.mdpi.com/article/10.3390/molecules29010145/s1, Supplementary Figure S1: Total ion current diagram of seven Lamiaceae plant hydrosols. (A) Thymus vulgaris; (B) Thymus mongolicus; (C) Mentha×piperita; (D) Melissa officinalis; (E) Rosmarinus officinali; (F) Salvia elegans; (G) Leonurus artemisia. Supplementary Table S1. Identified volatile metabolites of seven Lamiaceae plant hydrosols. Supplementary Table S2. Relative content (%) of differential metabolites of seven Lamiaceae plant hydrosols.
