# Supplementary material for: Investigation of Volatile Components and Assessment of Antioxidant Potential in Seven Lamiaceae Plant Hydrosols
Source: Molecules. 2023 Dec 26;29(1):145. doi: 10.3390/molecules29010145 (PMC10780048; doi:10.3390/molecules29010145)
Supplement: Supplementary file 1 [file molecules-29-00145-s001.zip › molecules-2781785-supplementary.pdf]

Supplementary Materials

Investigation of Volatile Components and Assessment of Antioxidant Potential in Seven Lamiaceae Plant Hydrosols

Supplementary Tables

Supplementary Table S1. Identified volatile metabolites of seven Lamiaceae plant hydrosols.

No.	Compound name	Formula	Relative content%						
			Tv HD	Tm HD	Mp HD	Mo HD	Ro HD	Se HD	La HD
1	Butanoic acid, 2-methyl-, methyl ester	C6H12O2	tr	nd	nd	nd	nd	nd	nd
2	1-Octen-3-ol	C8H16O	1.6135	1.9071	0.0273	nd	nd	nd	nd
3	Chloroacetic acid, cyclohexyl ester	C8H13ClO2	tr	nd	nd	nd	nd	nd	nd
4	Hexenyl tiglate, 4Z-	C11H18O2	tr	nd	nd	nd	nd	nd	nd
5	o-Cymene	C10H14	2.3984	0.3410	nd	nd	nd	nd	nd
6	Eucalyptol	C10H18O	3.6612	3.0541	4.2754	5.1455	6.9731	0.1632	2.7112
7	1H-2-Indenol, 2,3,4,5,6,7-hexahydro-1-(2-hydroxy-2-methylpropyl)	C13H22O2	tr	nd	nd	nd	nd	nd	nd
8	2,6-Octadien-1-ol, 2,7-dimethyl-	C10H18O	tr	nd	nd	nd	nd	nd	nd
9	Butanoic acid, 4-pentenyl ester	C9H16O2	0.1287	tr	nd	nd	nd	nd	nd
10	Benzene, 1-methyl-4-(1-methylethenyl)-	C10H12	0.0597	nd	nd	nd	0.0237	nd	nd
11	Linalool	C10H18O	4.8946	5.9509	0.2774	2.7061	6.3686	19.2715	0.3818
12	2,6-Octadien-1-ol, 3,7-dimethyl-, (Z)-	C10H18O	0.0443	nd	nd	nd	nd	nd	nd
13	7-Octen-4-ol, 2-methyl-6-methylene-, (S)-	C10H18O	0.0209	nd	nd	nd	nd	nd	nd
14	Cyclohexanol, 1-methyl-4-(1-methylethenyl)-, cis-	C10H18O	0.0684	nd	nd	nd	nd	nd	nd
15	2-Cyclohexen-1-ol, 1-methyl-4-(1-methylethyl)-, trans-	C10H18O	tr	nd	nd	nd	0.0170	nd	nd
16	2-Cyclohexen-1-ol, 1-methyl-4-(1-methylethyl)-, cis-	C10H18O	0.0235	0.0272	nd	nd	nd	nd	nd
17	Isopinocarveol	C10H16O	0.0510	nd	tr	nd	nd	nd	nd
18	1,2-15,16-Diepoxyhexadecane	C16H30O2	0.0970	nd	nd	nd	nd	nd	nd
19	3-Cyclohexene-1-carboxaldehyde, 1,3,4-trimethyl-	C10H16O	0.1502	nd	nd	nd	nd	0.0174	nd
20	Ethanol, 2-(9,12-octadecadienyloxy)-, (Z,Z)-	C20H38O2	0.0111	0.0352	nd	nd	nd	nd	0.1339
21	endo-Borneol	C10H18O	1.8083	2.0337	0.5643	nd	5.5855	nd	nd
22	3-Cyclohexen-1-ol, 4-methyl-1-(1-methylethyl)-, (R)-	C10H18O	3.0542	nd	nd	nd	2.0065	nd	3.0187
23	Butanoic acid, 3-hexenyl ester, (E)-	C10H18O2	0.3914	nd	nd	nd	nd	nd	nd
24	L-à-Terpineol	C10H18O	0.3081	nd	nd	nd	2.4627	nd	0.4924
25	Dihydroxanthin	C17H24O5	0.0536	nd	nd	nd	nd	nd	nd
26	Cyclohexanol, 5-methyl-2-(1-methylethenyl)-	C10H18O	tr	0.0118	nd	nd	tr	nd	nd
27	2-Cyclohexen-1-ol, 3-methyl-6-(1-methylethyl)-, cis-	C10H18O	0.0143	nd	nd	nd	nd	nd	nd
28	cis-p-mentha-1(7),8-dien-2-ol	C10H16O	0.0139	0.0203	nd	nd	0.0298	tr	tr
29	Isobornyl formate	C11H18O2	0.0437	nd	nd	nd	nd	0.4519	nd

30	Benzene, 2-methoxy-4-methyl-1-(1-methylethyl)-	C11H16O	6.2762	2.2405	nd	nd	nd	nd	nd
31	(-)-Carvone	C10H14O	1.7551	nd	0.2512	1.6204	nd	nd	nd
32	Geraniol	C10H18O	0.1222	0.3183	nd	1.4782	4.0724	nd	nd
33	Spiro[4.5]dec-6-en-8-one, 1,7-dimethyl-4-(1-methylethyl)-	C15H24O	tr	nd	nd	nd	nd	nd	tr
34	2(3H)-Naphthalenone, 4,4a,5,6,7,8-hexahydro-1-methoxy-	C11H16O2	tr	nd	nd	nd	nd	nd	nd
35	6-Octen-1-yn-3-ol, 3,7-dimethyl-	C10H16O	tr	nd	nd	nd	nd	nd	nd
36	Benzene, 1-methoxy-4-(1-propenyl)-, (Z)-	C10H12O	0.1426	nd	1.2398	nd	nd	nd	nd
37	Phenol, 2-methyl-5-(1-methylethyl)-	C10H14O	1.8560	1.8690	0.1807	nd	nd	tr	nd
38	Thymol	C10H14O	18.8404	19.5366	nd	1.3675	0.0692	5.3207	1.0919
39	2,6-Octadienoic acid, 3,7-dimethyl-, methyl ester, (Z)-	C11H18O2	tr	nd	nd	0.0666	nd	nd	nd
40	(-)-8-p-Menthen-2-yl, acetate, trans	C12H20O2	0.0195	nd	0.4261	nd	nd	nd	nd
41	2-Cyclohexen-1-one, 3-methyl-6-(1-methylethylidene)-	C10H14O	tr	nd	0.1066	nd	0.0452	nd	nd
42	Cyclopropane, 1-methoxy-2,2-dimethyl-3-(3,3-dimethyl-1-propynyl)-	C12H20O	tr	nd	nd	nd	nd	nd	nd
43	Phenol, 3-(1,1-dimethylethyl)-4-methoxy-	C11H16O2	0.0580	nd	nd	nd	nd	nd	nd
44	Phenol, 5-methyl-2-(1-methylethyl)-, acetate	C12H16O2	0.0748	0.4030	nd	nd	nd	nd	nd
45	3-Allyl-6-methoxyphenol	C10H12O2	0.0204	nd	0.0353	nd	0.0376	nd	0.0662
46	Geranyl acetate	C12H20O2	0.0233	0.1286	nd	0.1516	0.0742	tr	nd
47	Isobornyl propionate	C13H22O2	0.0233	0.0166	nd	nd	nd	nd	nd
48	Propanoic acid, 2-methyl-, 2-phenylethyl ester	C12H16O2	tr	nd	nd	nd	nd	nd	nd
49	2-Cyclopenten-1-one, 3-methyl-2-(2-pentenyl)-, (Z)-	C11H16O	tr	nd	0.0673	nd	0.0144	0.1150	nd
50	Methyleugenol	C11H14O2	0.0440	0.0631	0.0975	0.5505	0.4481	nd	nd
51	Benzene, 1,3,5-trimethoxy-	C9H12O3	0.0359	0.1483	0.0776	nd	nd	0.0645	nd
52	Propanoic acid, 2-methyl-, 1,7,7-trimethylbicyclo[2.2.1]hept-2-yl ester, exo-	C14H24O2	0.0121	nd	nd	nd	nd	nd	nd
53	Caryophyllene	C15H24	0.1823	nd	0.1308	nd	nd	nd	nd
54	Adamantane, 2-hydroperoxy-2-ethenyl-	C12H18O2	tr	nd	nd	nd	nd	nd	nd
55	Ethanone, 1-(2-hydroxy-4-methoxyphenyl)-	C9H10O3	0.2473	0.0840	0.4665	0.5262	0.0576	0.3548	nd
56	Humulene	C15H24	0.0178	nd	0.0500	nd	nd	nd	0.1541
57	(E)-á-Famesene	C15H24	tr	nd	0.0366	nd	nd	nd	nd
58	Cholestan-3-ol, 2-methylene-, (3á,5à)-	C28H48O	tr	nd	nd	nd	nd	nd	nd
59	2,6-Octadien-1-ol, 3,7-dimethyl-, propanoate, (Z)-	C13H22O2	0.0155	nd	nd	nd	nd	nd	nd
60	ç-Muurolene	C15H24	0.1161	nd	tr	nd	nd	nd	nd
61	8-Decene-3,5-dione, 2,4,6,9-tetramethyl-	C14H24O2	0.0296	nd	0.0914	nd	nd	nd	nd
62	N,N-dipropionylphenethylamine	C14H19NO2	tr	nd	nd	nd	nd	nd	nd
63	Phenethyl palmitate	C24H40O2	tr	nd	nd	nd	nd	nd	nd
64	Guaia-1(10),11-diene	C15H24	tr	nd	nd	nd	nd	0.0412	nd

65	Naphthalene, 1,2,4a,5,6,8a-hexahydro-4,7-dimethyl-1-(1-methylethyl)-,[1R-(1à,4aà,8aà)]-	C15H24	0.0144	nd	tr	nd	nd	nd	nd
66	Butanoic acid, 3-methyl-, 1,7,7-trimethylbicyclo[2.2.1]hept-2-yl ester, exo-	C15H26O2	tr	nd	nd	nd	nd	nd	nd
67	cis-Calamenene	C15H22	0.0498	nd	nd	nd	nd	nd	nd
68	1-Heptatriacotanol	C37H76O	0.0267	0.0247	nd	nd	nd	0.0150	nd
69	Tau-Cadinol acetate	C17H28O2	tr	nd	nd	nd	nd	nd	nd
70	(3R,5aS,9aR)-2,2,5a,9-Tetramethyl-3,4,5,5a,6,7-hexahydro-2H-3,9a-methanobenzo[b]oxepine	C15H24O	tr	nd	nd	nd	nd	nd	nd
71	Caryophyllene oxide	C15H24O	0.2775	0.1563	0.1349	1.2640	0.0480	2.4263	0.6687
72	p-Cymene-2,5-diol	C10H14O2	tr	nd	nd	nd	nd	0.1361	tr
73	Butanoic acid, 3,7-dimethyl-2,6-octadienyl ester, (E)-	C14H24O2	tr	nd	nd	nd	nd	nd	nd
74	6-epi-shyobunol	C15H26O	tr	nd	0.0351	nd	nd	nd	nd
75	(-)-Spathulenol	C15H24O	0.0140	0.1176	0.0640	2.2058	0.0718	2.8024	1.3419
76	1H-Cycloprop[e]azulen-4-ol, decahydro-1,1,4,7-tetramethyl-, [1aR-(1aà,4á,4aá,7à,7aá,7bà)]-	C15H26O	0.1746	nd	0.8540	nd	0.0282	1.6843	nd
77	(1S,3aS,4S,5S,7aR,8R)-5-Isopropyl-1,7a-dimethyloctahydro-1H-1,4-methanoinden-8-ol	C15H26O	tr	nd	0.0185	nd	nd	nd	0.0105
78	(1R,3E,7E,11R)-1,5,5,8-Tetramethyl-12-oxabicyclo[9.1.0]dodeca-3,7-diene	C15H24O	tr	nd	nd	nd	nd	nd	nd
79	(1R,7S,E)-7-Isopropyl-4,10-dimethylenecyclodec-5-enol	C15H24O	tr	0.0145	0.0164	0.0353	nd	0.0118	nd
80	Epicubenol	C15H26O	0.0603	0.0220	0.0715	nd	nd	nd	nd
81	2-((2S,4aR)-4a,8-Dimethyl-1,2,3,4,4a,5,6,7-octahydronaphthalen-2-yl)propan-2-ol	C15H26O	0.0558	nd	nd	nd	nd	nd	nd
82	Cubenol	C15H26O	tr	nd	nd	nd	nd	nd	nd
83	Docosanoic acid, 8,9-dihydroxy-, methyl ester	C23H46O4	0.0142	nd	nd	nd	nd	nd	nd
84	i-Propyl 5,8,11,14,17-eicosapentaenoate	C23H36O2	0.0104	nd	nd	nd	nd	nd	nd
85	.tau.-Cadinol	C15H26O	0.2888	nd	0.0725	0.6238	0.0326	0.3298	0.5787
86	(3S,3aR,3bR,4S,7R,7aR)-4-Isopropyl-3,7-dimethyloctahydro-1H-cyclopenta[1,3]cyclopropa[1,2]benzen-3-ol	C15H26O	tr	nd	nd	nd	nd	nd	tr
87	2-Naphthalenemethanol, 2,3,4,4a,5,6,7,8-octahydro-à,à,4a,8-tetramethyl-, [2R-(2à,4aá,8á)]-	C15H26O	tr	nd	nd	nd	nd	nd	nd
88	à-Cadinol	C15H26O	0.0308	nd	0.1305	nd	nd	0.1313	nd
89	Isoaromadendrene epoxide	C15H24O	0.0291	tr	0.0163	nd	nd	nd	nd
90	Benz[e]azulene-3,8-dione, 5-[(acetyloxy)methyl]-3a,4,6a,7,9,10,10a,10b-octahydro-3a,10a-dihydroxy-2,10-dimethyl-, (3aà,6aà,10á,10aá,10bá)-(+)-	C19H24O6	tr	nd	nd	nd	nd	nd	nd
91	Heptadecane	C17H36	tr	0.0798	0.0105	nd	nd	nd	nd
92	Benzene, 1-(1,1-dimethylethyl)-4-methoxy-	C11H16O	tr	nd	nd	nd	nd	nd	nd
93	Propanoic acid, 2-(3-acetoxy-4,4,14-trimethylandrost-8-en-17-yl)-	C27H42O4	tr	nd	nd	nd	nd	nd	0.0139
94	Terpinen-4-ol	C10H18O	nd	4.2203	nd	0.5528	tr	0.3680	0.1803
95	3-Octanone	C8H16O	nd	2.3914	0.0823	4.6015	nd	6.8098	0.5702
96	Benzene, 2-methoxy-1-methyl-4-(1-methylethyl)-	C11H16O	nd	1.0979	nd	nd	nd	nd	nd
97	α-Terpineol	C10H18O	nd	0.5004	nd	nd	nd	nd	nd

98	D-Allose	C6H12O6	nd	0.4867	nd	nd	nd	nd	nd
99	(2S,4R)-4-Methyl-2-(2-methylprop-1-en-1-yl)tetrahydro-2H-pyran	C10H18O	nd	0.3266	nd	3.5400	nd	0.1761	nd
100	Phenol, 2-methoxy-3-(2-propenyl)-	C10H12O2	nd	0.2331	nd	nd	nd	nd	0.0537
101	t-Cadinol	C15H26O	nd	0.1911	nd	nd	nd	nd	nd
102	(1R,2R,5S)-5-Methyl-2-(prop-1-en-2-yl)cyclohexanol	C10H18O	nd	0.1683	nd	12.7966	tr	nd	nd
103	1-Hepten-3-ol	C7H14O	nd	0.1114	nd	nd	nd	nd	nd
104	7-Benzofuranol, 2,3-dihydro-2,2-dimethyl-	C10H12O2	nd	0.1094	nd	nd	nd	nd	nd
105	3,4-Altrosan	C6H10O5	nd	0.0972	nd	nd	nd	nd	nd
106	Cyclohexanone, 2-(1-methylethylidene)-	C9H14O	nd	0.0604	nd	nd	nd	nd	nd
107	1,7,7-Trimethylbicyclo[2.2.1]heptan-2-ol	C10H18O	nd	0.0536	nd	nd	nd	nd	nd
108	2,6-Octadien-1-ol, 3,7-dimethyl-, acetate, (Z)-	C12H20O2	nd	0.0474	0.0273	0.0661	nd	nd	nd
109	Bicyclo[2.2.1]heptane-2,5-diol, 1,7,7-trimethyl-, (2-endo,5-exo)-	C10H18O2	nd	0.0471	nd	nd	nd	nd	nd
110	2-Cyclopenten-1-one, 2-(2-butenyl)-3-methyl-, (Z)-	C10H14O	nd	0.0468	nd	nd	nd	nd	nd
111	exo-2,7,7-trimethylbicyclo[2.2.1]heptan-2-ol	C10H18O	nd	0.0445	nd	nd	nd	tr	nd
112	3-Nonenoic acid	C9H16O2	nd	0.0438	nd	nd	nd	nd	nd
113	Cyclohexene, 6-butyl-1-nitro-	C10H17NO2	nd	0.0386	nd	nd	nd	nd	nd
114	6,6-Dimethyl-9-propenyl-1,4-dioxa-spiro[4.5]decane	C13H22O2	nd	0.0340	nd	nd	nd	nd	nd
115	3,5-Dimethoxy-4-hydroxyphenylacetic acid	C10H12O5	nd	0.0340	nd	nd	nd	nd	nd
116	3,5-Dimethoxy-4-hydroxytoluene	C9H12O3	nd	0.0303	nd	nd	nd	nd	nd
117	4,4'-(3,3'-Dinitro-4,4'-biphenylylenebisazo)diphenol	C24H16N6O6	nd	0.0301	nd	nd	nd	nd	nd
118	3,6-Octadien-1-ol, 3,7-dimethyl-, (Z)-	C10H18O	nd	0.0285	nd	0.1725	nd	nd	nd
119	trans-Z-α-Bisabolene epoxide	C15H24O	nd	0.0285	nd	nd	nd	nd	nd
120	Benzene, 1,2,3-trimethoxy-5-methyl-	C10H14O3	nd	0.0228	nd	nd	nd	nd	nd
121	1-Chlorosulfonyl-3-methyl-1-azaspiro[3.5]nonan-2-one	C9H14ClNO3S	nd	0.0222	nd	nd	nd	nd	nd
122	α-Cadinol	C15H26O	nd	0.0218	nd	nd	nd	nd	nd
123	1,6-Octadiene, 3-ethoxy-3,7-dimethyl-	C12H22O	nd	0.0182	nd	nd	nd	nd	nd
124	Butanamide, N-methyl-4-(methylthio)-2-(2,2-dimethylpropylidene)amino-	C11H22N2OS	nd	0.0173	nd	nd	nd	nd	nd
125	Oxime-, methoxy-phenyl-_	C8H9NO2	nd	0.0168	nd	nd	nd	nd	nd
126	1-(4-Methoxy-2-nitroanilino)-1-deoxy-a-d-arabinofuranose	C12H16N2O7	nd	0.0165	nd	nd	nd	nd	nd
127	Eicosane	C20H42	nd	0.0163	nd	nd	nd	nd	nd
128	Nerolidyl acetate	C17H28O2	nd	0.0157	nd	nd	nd	nd	nd
129	5α,7αH,10α-Eudesm-11-en-1α-ol	C15H26O	nd	0.0154	nd	nd	nd	nd	nd
130	2,2,4-Trimethyl-1,3-pentanediol diisobutyrate	C16H30O4	nd	0.0148	nd	nd	nd	nd	nd
131	Benzene, (3-octylundecyl)-	C25H44	nd	0.0146	nd	nd	nd	nd	nd
132	Hexadecanoic acid, 1a,2,5,5a,6,9,10,10a-octahydro-5a-hydroxy-4-(hydroxymethyl)-1,1,7,9-tetramethyl-6,11-dioxo-1H-2,8a-	C36H56O6	nd	0.0139	nd	nd	nd	nd	nd

	methanocyclopenta[a]cyclopropa[e]cyclodecen-5-yl ester, [1aR-(1aα,2α,5α,5aα,8aα,9α,10aα)]-								
133	2-Phenylacetamide, N-(1-phenyl-2-propyl)-	C17H19NO	nd	0.0139	nd	nd	nd	nd	nd
134	Ethanol, 2-(3,3-dimethylcyclohexylidene)-, (Z)-	C10H18O	nd	0.0132	nd	nd	0.2363	nd	nd
135	Butanoic acid, 1,7,7-trimethylbicyclo[2.2.1]hept-2-yl ester, endo-	C14H24O2	nd	0.0119	nd	nd	nd	nd	nd
136	Pentadecane, 3-methyl-	C16H34	nd	0.0117	nd	nd	nd	nd	nd
137	2-Propanone, 1-(4-hydroxy-3-methoxyphenyl)-	C10H12O3	nd	0.0111	nd	nd	nd	nd	nd
138	10-Hydroxydecanoic acid, methyl ester	C11H22O3	nd	0.0109	nd	nd	nd	nd	nd
139	2,4-Diazapentane, N,N'-dimethyl-3,3-bis[cycloazapropyl]-	C9H20N4	nd	0.0106	nd	nd	nd	nd	nd
140	2,5-Octadecadiynoic acid, methyl ester	C19H30O2	nd	tr	nd	0.4747	nd	tr	nd
141	Heptacosane, 1-chloro-	C27H55Cl	nd	tr	nd	nd	nd	nd	nd
142	1,5-Hexadiene, 3,3,4,4-tetrafluoro-	C6H6F4	nd	tr	nd	nd	nd	nd	nd
143	(E)-α-Famesene	C15H24	nd	tr	nd	nd	nd	nd	nd
144	2,5,5,8a-Tetramethyl-3,5,6,7,8,8a-hexahydro-2H-naphthalen-1-one	C14H22O	nd	tr	nd	nd	nd	nd	nd
145	Heptadecane, 9-hexyl-	C23H48	nd	tr	nd	nd	nd	nd	nd
146	2,7-Diphenyl-1,6-dioxopyridazino[4,5:2',3']pyrrolo[4',5'-d]pyridazine	C20H13N5O2	nd	tr	nd	nd	nd	nd	nd
147	(E)-15,16-Dinorlabda-8(17),12-dien-14-al	C18H28O	nd	tr	nd	nd	nd	nd	nd
148	Benzenemethanol, 4-methyl-α-(1-methyl-2-propenyl)-, (R*,R*)-	C12H16O	nd	tr	nd	nd	nd	nd	nd
149	3,5-Heptadienal, 2-ethylidene-6-methyl-	C10H14O	nd	tr	nd	nd	nd	nd	0.0433
150	9-Nonadecene	C19H38	nd	tr	nd	nd	nd	nd	nd
151	α-D-Mannofuranoside, farnesyl-	C21H36O6	nd	tr	nd	nd	nd	nd	nd
152	Methanone, (phenyl)(4-pyridyl)-, 2-tolylsulfonylhydrazone	C19H17N3O2S	nd	tr	nd	nd	nd	nd	nd
153	2,5,5,8a-Tetramethyl-4-methylene-6,7,8,8a-tetrahydro-4H,5H-chromen-4a-yl hydroperoxide	C14H22O3	nd	tr	nd	nd	nd	nd	nd
154	2-Propen-1-ol, 3-(2,6,6-trimethyl-1-cyclohexen-1-yl)-	C12H20O	nd	tr	nd	nd	nd	nd	nd
155	Dibutyl phthalate	C16H22O4	nd	tr	tr	nd	nd	nd	nd
156	4-isopropyl-1,6-dimethyl-1,2,3,4-tetrahydronaphthalene	C15H22	nd	tr	nd	nd	nd	nd	nd
157	Octadecane, 3-ethyl-5-(2-ethylbutyl)-	C26H54	nd	tr	nd	0.0975	tr	nd	nd
158	Phenol, 2-methoxy-6-(1-propenyl)-	C10H12O2	nd	tr	nd	nd	nd	nd	nd
159	Ethanol, 2-(9-octadecenyloxy)-, (Z)-	C20H40O2	nd	tr	nd	nd	nd	nd	nd
160	1-Nonadecanamine, N,N-dimethyl-	C21H45N	nd	tr	nd	nd	nd	nd	nd
161	Ethanone, 1-(4-hydroxy-3,5-dimethoxyphenyl)-	C10H12O4	nd	tr	nd	nd	nd	nd	nd
162	9,10-Secocholesta-5,7,10(19)-triene-3,24,25-triol, (3α,5Z,7E)-	C27H44O3	nd	tr	nd	nd	nd	nd	nd
163	α-acorenol	C15H26O	nd	tr	nd	nd	nd	nd	nd
164	Perhydroindene-4-carboxylic acid, 6-acetoxy-2,3-epoxy-1,1-epoxymethyl-3a-hydroxy-5-isopropenyl-7a-methyl-7-oxo-, methyl ester	C18H22O8	nd	tr	nd	nd	nd	nd	nd
165	Cholestan-3-one, cyclic 1,2-ethanediyl aetal, (5α)-	C29H50O2	nd	tr	nd	nd	nd	nd	nd

166	Cyclohexanone, 2-(2-butynyl)-	C10H14O	nd	tr	nd	nd	nd	nd	nd
167	9-Octadecenoic acid, (2-phenyl-1,3-dioxolan-4-yl)methyl ester, cis-	C28H44O4	nd	tr	nd	nd	nd	nd	nd
168	i-Propyl 12-methyl-tridecanoate	C17H34O2	nd	tr	nd	nd	nd	nd	nd
169	Cholestan-3-ol, 2-methylene-, (3α,5α)-	C28H48O	nd	tr	nd	nd	nd	nd	nd
170	Methyl 6,7-di-O-acetyl-2,3,4-tri-O-methyl-α-glycero-D-glucoheptopyranoside	C15H26O9	nd	tr	nd	nd	nd	nd	nd
171	Bicyclo[3.1.0]hex-2-ene, 4-methylene-1-(1-methylethyl)-	C10H14	nd	tr	nd	nd	0.0236	nd	nd
172	Bicyclo[3.1.0]hex-3-en-2-ol, 2-methyl-5-(1-methylethyl)-, (1α,2α,5α)-	C10H16O	nd	tr	nd	nd	nd	nd	nd
173	2-Nonadecanone 2,4-dinitrophenylhydrazine	C25H42N4O4	nd	tr	nd	tr	nd	nd	nd
174	7-epi-cis-sesquisabinene hydrate	C15H26O	nd	tr	tr	nd	nd	nd	tr
175	Oleic acid, 3-(octadecyloxy)propyl ester	C39H76O3	nd	tr	nd	nd	nd	nd	nd
176	Dodecane, 5,8-diethyl-	C16H34	nd	tr	nd	nd	nd	nd	nd
177	Hexadecanoic acid, ethyl ester	C18H36O2	nd	tr	nd	nd	nd	nd	nd
178	9-Hexadecenoic acid, 9-octadecenyl ester, (Z,Z)-	C34H64O2	nd	tr	nd	nd	nd	nd	nd
179	4-(3,5-Dimethoxydecyl)-1,2-dimethoxybenzene	C20H34O4	nd	tr	nd	nd	nd	nd	nd
180	Carvone	C10H14O	nd	nd	26.8502	nd	nd	nd	1.7867
181	cis-Dihydrocarvone	C10H16O	nd	nd	10.4092	nd	nd	nd	nd
182	Cyclohexanone, 2-methyl-5-(1-methylethenyl)-, trans-	C10H16O	nd	nd	0.7362	nd	nd	nd	nd
183	Bicyclo[3.1.0]hexan-3-ol, 4-methyl-1-(1-methylethyl)-	C10H18O	nd	nd	0.3709	nd	nd	nd	nd
184	Citral	C10H16O	nd	nd	0.2677	nd	nd	nd	nd
185	trans-Carveol	C10H16O	nd	nd	0.2468	nd	nd	nd	nd
186	Isopimara-9(11),15-diene	C20H32	nd	nd	0.2016	nd	nd	nd	nd
187	(-)-á-Bourbonene	C15H24	nd	nd	0.1210	nd	nd	nd	nd
188	Bicyclo[3.1.0]hexan-3-one, 4-methyl-1-(1-methylethyl)-	C10H16O	nd	nd	0.1172	nd	nd	nd	nd
189	Ketone, 3aá,4,5,6,7,7a-hexahydro-7aá-methyl-1à-indanyl methyl	C12H20O	nd	nd	0.0993	nd	nd	nd	nd
190	1-Isopropyl-4,7-dimethyl-1,2,3,5,6,8a-hexahydronaphthalene	C15H24	nd	nd	0.0804	nd	nd	nd	0.3593
191	isoledene	C15H24	nd	nd	0.0631	nd	nd	nd	nd
192	(1R,4aR,4bS,7S,10aR)-1,4a,7-Trimethyl-7-vinyl-1,2,3,4,4a,4b,5,6,7,8,10,10a-dodecahydrophenanthrene-1-carbaldehyde	C20H30O	nd	nd	0.0405	nd	nd	nd	nd
193	Hibaene	C20H32	nd	nd	0.0381	nd	nd	nd	nd
194	Limonene oxide, cis-	C10H16O	nd	nd	0.0339	nd	nd	nd	nd
195	1-Nonen-3-ol	C9H18O	nd	nd	0.0329	nd	nd	nd	nd
196	2,4-Di-tert-butylphenol	C14H22O	nd	nd	0.0310	nd	tr	nd	nd
197	1H-Cycloprop[e]azulene, 1a,2,3,5,6,7,7a,7b-octahydro-1,1,4,7-tetramethyl-, [1aR-(1aà,7à,7aá,7bà)]-	C15H24	nd	nd	0.0283	nd	nd	nd	nd
198	trans-Carveyl acetate	C12H18O2	nd	nd	0.0265	nd	nd	nd	nd
199	cis-Muurola-4(15),5-diene	C15H24	nd	nd	0.0263	nd	nd	nd	nd
200	5,9-Dodecadien-2-one, 6,10-dimethyl-, (E,E))-	C14H24O	nd	nd	0.0252	nd	nd	nd	nd

201	Germacrene D	C15H24	nd	nd	0.0251	nd	nd	nd	nd
202	Tetradecane, 2,6,10-trimethyl-	C17H36	nd	nd	0.0249	nd	nd	nd	nd
203	1H-2,8a-Methanocyclopenta[a]cyclopropa[e]cyclodecen-11-one, 1a,2,5,5a,6,9,10,10a-octahydro-5,5a,6-trihydroxy-1,4-bis(hydroxymethyl)-1,7,9-trimethyl-, [1S-(1à,1aà,2à,5á,5aá,6á,8aà,9à,10aà)]-	C20H28O6	nd	nd	0.0182	nd	0.0125	nd	nd
204	Selin-6-en-4à-ol	C15H26O	nd	nd	0.0152	nd	nd	nd	nd
205	10,10-Dimethyl-2,6-dimethylenebicyclo[7.2.0]undecan-5á-ol	C15H24O	nd	nd	0.0116	nd	nd	nd	nd
206	(1S,4aR,8aS)-1-Isopropyl-7-methyl-4-methylene-1,2,3,4,4a,5,6,8a-octahydronaphthalene	C15H24	nd	nd	0.0115	nd	nd	nd	nd
207	Bicyclo[3.1.0]hexan-3-ol, 4-methyl-1-(1-methylethyl)-, (1à,3á,4á,5à)-	C10H18O	nd	nd	tr	nd	nd	nd	nd
208	Doconexent	C22H32O2	nd	nd	tr	nd	nd	0.0489	nd
209	Melezitose	C18H32O16	nd	nd	tr	nd	nd	nd	nd
210	(Z)-Hex-3-enyl (E)-2-methylbut-2-enoate	C11H18O2	nd	nd	tr	nd	nd	nd	nd
211	á-copaene	C15H24	nd	nd	tr	nd	nd	0.0256	nd
212	1,4-Methanoazulen-3-ol, decahydro-1,5,5,8a-tetramethyl-, [1S-(1à,3á,3aá,4à,8aá)]-	C15H26O	nd	nd	tr	nd	tr	nd	nd
213	Carvone oxide, cis-	C10H14O2	nd	nd	tr	nd	nd	nd	nd
214	1H-Cycloprop[e]azulene, decahydro-1,1,7-trimethyl-4-methylene-	C15H24	nd	nd	tr	nd	nd	nd	nd
215	(S)-2,2,6-Trimethyl-6-((S)-4-methylcyclohex-3-en-1-yl)dihydro-2H-pyran-3(4H)-one	C15H24O2	nd	nd	tr	0.2577	0.0359	nd	0.4357
216	Furan, 2,5-diethyltetrahydro-	C8H16O	nd	nd	tr	nd	nd	nd	nd
217	3-Nonanol	C9H20O	nd	nd	tr	nd	nd	nd	nd
218	1-Naphthalenol, 1,2,3,4,4a,7,8,8a-octahydro-1,6-dimethyl-4-(1-methylethyl)-, [1S-(1à,4à,4aá,8aá)]-	C15H26O	nd	nd	tr	nd	nd	nd	nd
219	1-Hexadecanol, 2-methyl-	C17H36O	nd	nd	tr	nd	nd	nd	nd
220	8,11,14-Eicosatrienoic acid, methyl ester, (Z,Z,Z)-	C21H36O2	nd	nd	tr	nd	nd	nd	nd
221	1H-Cyclopropa[3,4]benz[1,2-e]azulene-5,7b,9,9a-tetrol, 1a,1b,4,4a,5,7a,8,9-octahydro-3-(hydroxymethyl)-1,1,6,8-tetramethyl-, 5,9,9a-triacetate, [1aR-(1aà,1bá,4aá,5á,7aà,7bà,8à,9á,9aà)]-	C26H36O8	nd	nd	tr	nd	nd	nd	nd
222	Benzoic acid, 4-methoxy-, methyl ester	C9H10O3	nd	nd	tr	nd	nd	nd	nd
223	2,6-Dimethyl-1,3,5,7-octatetraene, E,E-	C10H14	nd	nd	tr	nd	nd	nd	nd
224	Globulol	C15H26O	nd	nd	tr	nd	nd	nd	nd
225	Hexadecane, 1,1-bis(dodecyloxy)-	C40H82O2	nd	nd	tr	nd	nd	nd	nd
226	13-Heptadecyn-1-ol	C17H32O	nd	nd	tr	nd	nd	nd	nd
227	Aspidospermidin-17-ol, 1-acetyl-19,21-epoxy-15,16-dimethoxy-	C23H30N2O5	nd	nd	tr	nd	nd	nd	nd
228	Z,Z,Z-4,6,9-Nonadecatriene	C19H34	nd	nd	tr	nd	nd	nd	nd
229	Octadecane, 6-methyl-	C19H40	nd	nd	tr	nd	nd	nd	nd
230	1H-Indene, 3-(bromomethyl)-1,1-dimethyl-	C12H13Br	nd	nd	tr	nd	nd	nd	nd
231	Cyclohexanone, 2,2-dimethyl-5-(3-methyloxiranyl)-, [2à(R*),3à]-(.+-.)-	C11H18O2	nd	nd	nd	0.0668	nd	nd	nd
232	10-Undecyn-1-ol	C11H20O	nd	nd	nd	1.8064	nd	nd	nd

233	Z,Z-4,16-Octadecadien-1-ol acetate	C20H36O2	nd	nd	nd	0.3050	nd	nd	nd
234	4-Hexen-1-ol, 5-methyl-2-(1-methylethenyl)-	C10H18O	nd	nd	nd	0.7919	nd	nd	nd
235	Terpineol	C10H18O	nd	nd	nd	0.4324	nd	0.3394	nd
236	Cyclohexanol, 2-methyl-5-(1-methylethenyl)-, (1à,2à,5á)-	C10H18O	nd	nd	nd	0.4048	nd	0.3007	nd
237	Bicyclo[5.1.0]octane, 8-(1-methylethylidene)-	C11H18	nd	nd	nd	0.3883	nd	nd	nd
238	3-Cyclohexene-1-methanol, 5-hydroxy-à,à,4-trimethyl-, (1S-trans)-	C10H18O2	nd	nd	nd	0.0393	nd	nd	nd
239	Paromomycin	C23H45N5O14	nd	nd	nd	0.2843	nd	nd	nd
240	Longipinene epoxide	C15H24O	nd	nd	nd	tr	nd	nd	nd
241	2,6-Octadienal, 3,7-dimethyl-, (Z)-	C10H16O	nd	nd	nd	0.6797	nd	1.6831	nd
242	1-Methyl-7-azabicyclo[4.1.0]hepta-2,4-diene-7-carboxylic acid, 3,17-diacetoxy-4,4,10,13-tetramethylhexadecahydrocyclopenta[a]phenanthrene	C33H47NO6	nd	nd	nd	0.1429	nd	nd	nd
243	(S)-(-)-Citronellic acid, methyl ester	C11H20O2	nd	nd	nd	1.3595	nd	nd	nd
244	2-Pentene, 4-methyl-, (Z)-	C6H12	nd	nd	nd	0.3179	nd	nd	nd
245	cis-Z-à-Bisabolene epoxide	C15H24O	nd	nd	nd	0.1445	0.0148	nd	nd
246	4-Hexen-1-ol, 5-methyl-2-(1-methylethenyl)-, acetate	C12H20O2	nd	nd	nd	0.6558	nd	nd	nd
247	.psi.,.psi.-Carotene, 1,1',2,2'-tetrahydro-1,1'-dimethoxy-	C42H64O2	nd	nd	nd	0.0366	nd	nd	nd
248	cis-5,8,11,14,17-Eicosapentaenoic acid	C20H30O2	nd	nd	nd	tr	nd	nd	nd
249	Benzene, 1,2-dimethoxy-4-propenyl-, (Z)-	C11H14O2	nd	nd	nd	tr	nd	0.2755	nd
250	(1aR,4S,4aR,7R,7aS,7bS)-1,1,4,7-Tetramethyldecahydro-1H-cyclopropa[e]azulen-4-ol	C15H26O	nd	nd	nd	0.3467	nd	nd	0.5315
251	Propanoic acid, 2-methyl-, (dodecahydro-6a-hydroxy-9a-methyl-3-methylene-2,9-dioxoazuleno[4,5-b]furan-6-yl)methyl ester, [3aS-(3aà,6á,6aà,9aá,9bà)]-	C19H26O6	nd	nd	nd	0.0386	nd	nd	0.0357
252	Brefeldin A	C16H24O4	nd	nd	nd	0.0307	nd	nd	nd
253	11,11-Dimethyl-4,8-dimethylenebicyclo[7.2.0]undecan-3-ol	C15H24O	nd	nd	nd	0.0626	tr	0.2944	nd
254	.tau.-Muurolol	C15H26O	nd	nd	nd	0.8074	nd	nd	nd
255	Bicyclo[3.1.1]hept-3-en-2-one, 4,6,6-trimethyl-, (1S)-	C10H14O	nd	nd	nd	nd	9.7675	nd	nd
256	Bicyclo[2.2.1]heptan-2-one, 1,7,7-trimethyl-, (1S)-	C10H16O	nd	nd	nd	nd	4.0883	nd	1.1876
257	Bicyclo[2.2.1]heptan-2-ol, 1,7,7-trimethyl-, acetate, (1S-endo)-	C12H20O2	nd	nd	nd	nd	1.5838	nd	nd
258	Bicyclo[3.1.1]heptan-3-one, 2,6,6-trimethyl-, (1à,2á,5à)-	C10H16O	nd	nd	nd	nd	1.4356	nd	nd
259	6-Octen-1-ol, 3,7-dimethyl-, (R)-	C10H20O	nd	nd	nd	nd	1.2039	nd	0.6678
260	Bicyclo[3.1.1]hept-2-ene-2-methanol, 6,6-dimethyl-	C10H16O	nd	nd	nd	nd	0.6757	nd	nd
261	Bicyclo[3.1.1]heptane-2-methanol, 6,6-dimethyl-, acetate	C12H20O2	nd	nd	nd	nd	0.6360	nd	nd
262	Pinocarvone	C10H14O	nd	nd	nd	nd	0.5421	nd	nd
263	2-Cyclohexen-1-one, 3-methyl-6-(1-methylethenyl)-, (S)-	C10H14O	nd	nd	nd	nd	0.3344	nd	nd
264	2H-Pyran, tetrahydro-4-methyl-2-(2-methyl-1-propenyl)-	C10H18O	nd	nd	nd	nd	0.1276	1.4923	0.0615
265	4,7,7-Trimethylbicyclo[4.1.0]hept-3-en-2-one	C10H14O	nd	nd	nd	nd	0.1158	nd	3.2877
266	Bicyclo[3.1.1]heptan-3-ol, 6,6-dimethyl-2-methylene-, [1S-(1à,3à,5à)]-	C10H16O	nd	nd	nd	nd	0.0655	nd	nd

267	3,9-Epoxy-p-mentha-1,8(10)-diene	C10H14O	nd	nd	nd	nd	0.0645	nd	nd
268	3,6-Octadienoic acid, 3,7-dimethyl-, methyl ester, (Z)-	C11H18O2	nd	nd	nd	nd	0.0609	nd	nd
269	Bisabolol oxide B	C15H26O2	nd	nd	nd	nd	0.0588	nd	0.9618
270	1-Isopropenyl-3-propenylcyclopentane	C11H18	nd	nd	nd	nd	0.0505	nd	nd
271	Bornyl acetate	C12H20O2	nd	nd	nd	nd	0.0473	nd	nd
272	trans-Chrysanthenol	C10H16O	nd	nd	nd	nd	0.0455	nd	0.0347
273	7-Propylidene-bicyclo[4.1.0]heptane	C10H16	nd	nd	nd	nd	0.0374	nd	nd
274	á-Pinene	C10H16	nd	nd	nd	nd	0.0318	nd	nd
275	Cyclopentane, 1-acetoxymethyl-3-isopropenyl-2-methyl-	C12H20O2	nd	nd	nd	nd	0.0264	nd	nd
276	3-Methyl-2-(2-methyl-2-butenyl)-furan	C10H14O	nd	nd	nd	nd	0.0213	nd	nd
277	(1S,4R,5R)-1,3,3-Trimethyl-2-oxabicyclo[2.2.2]octan-5-yl acetate	C12H20O3	nd	nd	nd	nd	0.0204	nd	nd
278	trans-Shisool	C10H18O	nd	nd	nd	nd	0.0191	nd	nd
279	Myrtenyl acetate	C12H18O2	nd	nd	nd	nd	0.0172	nd	nd
280	á-Ocimene	C10H16	nd	nd	nd	nd	0.0158	nd	nd
281	5-Hepten-2-one, 6-methyl-	C8H14O	nd	nd	nd	nd	tr	nd	nd
282	Cyclohexane, 1-methyl-2,4-bis(1-methylethenyl)-	C13H22	nd	nd	nd	nd	tr	nd	nd
283	2H-Pyran-3-ol, tetrahydro-2,2,6-trimethyl-6-(4-methyl-3-cyclohexen-1-yl)-, [3S-[3à,6à(R*)]]-	C15H26O2	nd	nd	nd	nd	tr	nd	0.0307
284	Geranyl vinyl ether	C12H20O	nd	nd	nd	nd	tr	0.0648	nd
285	Bicyclo[2.2.1]hept-2-ene, 1,7,7-trimethyl-	C10H16	nd	nd	nd	nd	tr	nd	nd
286	1,5,5-Trimethyl-6-methylene-cyclohexene	C10H16	nd	nd	nd	nd	tr	nd	nd
287	Isopulegol	C10H18O	nd	nd	nd	nd	tr	nd	nd
288	Erythro-9,10-dihydroxyoctadecanoic acid	C18H36O4	nd	nd	nd	nd	tr	nd	nd
289	Bicyclo[3.1.1]hept-3-en-2-one, 4,6,6-trimethyl-	C10H14O	nd	nd	nd	nd	tr	nd	nd
290	1,6,10-Dodecatrien-3-ol, 3,7,11-trimethyl-, (E)-	C15H26O	nd	nd	nd	nd	tr	nd	nd
291	4-Hydroxy-1,2,5-trimethyl-4-piperidinecarbonitrile, (2à,4à,5á)-	C9H16N2O	nd	nd	nd	nd	tr	nd	nd
292	3-Caren-10-al	C10H14O	nd	nd	nd	nd	tr	nd	nd
293	Ethanol	C2H6O	nd	nd	nd	nd	tr	nd	nd
294	trans-Verbenol	C10H16O	nd	nd	nd	nd	tr	0.1292	tr
295	Benzenemethanol, 4-methyl-à-(1-methyl-2-propenyl)-, (R*,R*)-	C12H16O	nd	nd	nd	nd	tr	nd	nd
296	Isospathulenol	C15H24O	nd	nd	nd	nd	tr	nd	nd
297	Di-epi-1,10-cubenol	C15H26O	nd	nd	nd	nd	tr	nd	nd
298	Alloaromadendrene oxide-(1)	C15H24O	nd	nd	nd	nd	tr	nd	nd
299	Butanoic acid, 2-hydroxy-3-methyl-4-(phenylmethoxy)-, [S-(R*,R*)]-	C12H16O4	nd	nd	nd	nd	tr	nd	nd
300	(Z)-2,6-Dimethylocta-2,5,7-trien-4-one	C10H14O	nd	nd	nd	nd	tr	nd	nd
301	Bicyclo[3.1.1]hept-3-en-2-ol, 4,6,6-trimethyl-, [1S-(1à,2á,5à)]-	C10H16O	nd	nd	nd	nd	tr	nd	nd

302	2,6-Octadienal, 3,7-dimethyl-, (E)-	C10H16O	nd	nd	nd	nd	nd	2.6581	nd
303	17-Octadecynoic acid	C18H32O2	nd	nd	nd	nd	nd	0.4751	nd
304	Geranyl formate	C11H18O2	nd	nd	nd	nd	nd	0.2418	nd
305	Ledene oxide-(II)	C15H24O	nd	nd	nd	nd	nd	0.1469	nd
306	Caryophylla-4(12),8(13)-dien-5à-ol	C15H24O	nd	nd	nd	nd	nd	0.1361	nd
307	Isobornyl thiocyanoacetate	C13H19NO2S	nd	nd	nd	nd	nd	0.1064	nd
308	Cyclohexane, 1,1'-dodecylidenebis[4-methyl-	C26H50	nd	nd	nd	nd	nd	0.0853	nd
309	6,10-Dodecadien-1-yn-3-ol, 3,7,11-trimethyl-	C15H24O	nd	nd	nd	nd	nd	0.0760	nd
310	Aromadendrene, dehydro-	C15H22	nd	nd	nd	nd	nd	0.0653	nd
311	Eugenol	C10H12O2	nd	nd	nd	nd	nd	0.0542	2.7807
312	3,6-Octadienal, 3,7-dimethyl-	C10H16O	nd	nd	nd	nd	nd	0.0537	nd
313	2H-Pyran, 3,6-dihydro-4-methyl-2-(2-methyl-1-propenyl)-	C10H16O	nd	nd	nd	nd	nd	0.0441	nd
314	(-)-Globulol	C15H26O	nd	nd	nd	nd	nd	0.0334	nd
315	(3R,3aR,3bR,4S,7R,7aR)-4-Isopropyl-3,7-dimethyloctahydro-1H-cyclopenta[1,3]cyclopropa[1,2]benzen-3-ol	C15H26O	nd	nd	nd	nd	nd	0.0318	nd
316	9,17-Octadecadienal, (Z)-	C18H32O	nd	nd	nd	nd	nd	0.0282	nd
317	Methyl 4,7,10,13-hexadecatetraenoate	C17H26O2	nd	nd	nd	nd	nd	0.0243	nd
318	Cholestan-3-one, cyclic 1,2-ethanediyl aetal, (5á)-	C29H50O2	nd	nd	nd	nd	nd	0.0210	nd
319	cis-Verbenol	C10H16O	nd	nd	nd	nd	nd	0.0196	nd
320	5-Hepten-3-yn-2-ol, 6-methyl-5-(1-methylethyl)-	C11H18O	nd	nd	nd	nd	nd	0.0181	nd
321	à-Methyl-à-[4-methyl-3-pentenyl]oxiranemethanol	C10H18O2	nd	nd	nd	nd	nd	0.0173	nd
322	Eicosane, 10-methyl-	C21H44	nd	nd	nd	nd	nd	0.0157	nd
323	1H-3a,7-Methanoazulene, octahydro-1,4,9,9-tetramethyl-	C15H26	nd	nd	nd	nd	nd	0.0143	nd
324	Dimethyl sulfide	C2H6S	nd	nd	nd	nd	nd	0.0128	nd
325	2-Buten-1-one, 1-(2,6,6-trimethyl-1-cyclohexen-1-yl)-	C13H20O	nd	nd	nd	nd	nd	0.0121	nd
326	Bicyclo[3.1.1]heptan-2-ol, 2,6,6-trimethyl-	C10H18O	nd	nd	nd	nd	nd	tr	nd
327	4aH-cycloprop[e]azulen-4a-ol, decahydro-1,1,4,7-tetramethyl-	C15H26O	nd	nd	nd	nd	nd	tr	nd
328	5-Isopropyl-2-methylbicyclo[3.1.0]hexan-2-ol #	C10H18O	nd	nd	nd	nd	nd	tr	nd
329	Aromadendrene oxide-(2)	C15H24O	nd	nd	nd	nd	nd	tr	nd
330	10,12-Octadecadiynoic acid	C18H28O2	nd	nd	nd	nd	nd	tr	nd
331	5aH-3a,12-Methano-1H-cyclopropa[5',6']cyclodeca[1',2':1,5]cyclopenta[1,2-d][1,3]dioxol-13-one, 1a,2,3,9,12,12a-hexahydro-9-hydroxy-10-(hydroxymethyl)-1,1,3,5,7,7-hexamethyl-, [1aR-(1aà,3à,3aà,5aà,8aR*,9á,12à,12aà)]-	C23H32O5	nd	nd	nd	nd	nd	tr	nd
332	Bicyclo[7.2.0]undec-4-ene, 4,11,11-trimethyl-8-methylene-,[1R-(1R*,4Z,9S*)]-	C15H24	nd	nd	nd	nd	nd	tr	3.7460
333	n-Nonenylsuccinic anhydride	C13H20O3	nd	nd	nd	nd	nd	tr	nd
334	7-Oxabicyclo[4.1.0]heptane, 1-methyl-4-(2-methyloxiranyl)-	C10H16O2	nd	nd	nd	nd	nd	tr	nd

335	9,10-Secocholesta-5,7,10(19)-triene-3,24,25-triol, (3á,5Z,7E)-	C27H44O3	nd	nd	nd	nd	nd	tr	0.0125
336	Dodecanoic acid, 1a,2,5,5a,6,9,10,10a-octahydro-5,5a-dihydroxy-4-(hydroxymethyl)-1,1,7,9-tetramethyl-11-oxo-1H-2,8a-methanocyclopenta[a]cyclopropa[e]cyclodecen-6-yl ester, [1aR-(1aà,2à,5á,5aá,6á,8aà,9à,10aà)]-	C32H50O6	nd	nd	nd	nd	nd	tr	nd
337	1b,5,5,6a-Tetramethyl-octahydro-1-oxa-cyclopropa[a]inden-6-one	C13H20O2	nd	nd	nd	nd	nd	tr	nd
338	Ethanethiol	C2H6S	nd	nd	nd	nd	nd	tr	nd
339	Thujone	C10H16O	nd	nd	nd	nd	nd	nd	9.2938
340	1,2,4,5-Tetrazine	C2H2N4	nd	nd	nd	nd	nd	nd	1.8961
341	1-Hydroxybicyclo[2.2.2]oct-5-en-2-yl, methyl ketone	C10H14O2	nd	nd	nd	nd	nd	nd	1.6909
342	(1R,5S,6R)-2,7,7-Trimethylbicyclo[3.1.1]hept-2-en-6-yl acetate	C12H18O2	nd	nd	nd	nd	nd	nd	1.6581
343	2-Cyclohexen-1-one, 4-hydroxy-3,5,5-trimethyl-4-(3-methyl-1,3-butadienyl)-, [S-(E)]-	C14H20O2	nd	nd	nd	nd	nd	nd	0.8519
344	6,7-Isoquinolinediol, 1,2,3,4-tetrahydro-2-methyl-	C10H13NO2	nd	nd	nd	nd	nd	nd	0.8328
345	2,4,6-Decatrienoic acid, 1a,2,5,5a,6,9,10,10a-octahydro-5,5a-dihydroxy-4-(hydroxymethyl)-1,1,7,9-tetramethyl-11-oxo-1H-2,8a-methanocyclopenta[a]cyclopropa[e]cyclodecen-6-yl ester, [1aR-(1aà,2à,5á,5aá,6á,8aà,9à,10aà)]-	C30H40O6	nd	nd	nd	nd	nd	nd	0.6222
346	Galactonic phenylhydrazide	C12H18N2O6	nd	nd	nd	nd	nd	nd	0.5114
347	Cyclohexanol, 2-methyl-5-(1-methylethenyl)-	C10H18O	nd	nd	nd	nd	nd	nd	0.4844
348	Octadecanal, 2-bromo-	C18H35BrO	nd	nd	nd	nd	nd	nd	0.4528
349	à-Cubebene	C15H24	nd	nd	nd	nd	nd	nd	0.4433
350	4-[4-(2-Methoxyphenyl)-1H-pyrazol-3-yl]benzene-1,3-diol	C16H14N2O3	nd	nd	nd	nd	nd	nd	0.4106
351	Nonane, 2,2,4,4,6,8,8-heptamethyl-	C16H34	nd	nd	nd	nd	nd	nd	0.3141
352	8-Oxabicyclo[4.3.0]nonane, 7,9-dimethyl-	C10H18O	nd	nd	nd	nd	nd	nd	0.3096
353	2-(2,6,6-Trimethylcyclohex-1-enyl)cyclopropanecarboxylic acid, methyl ester	C14H22O2	nd	nd	nd	nd	nd	nd	0.3071
354	1,1,4a-Trimethyl-5,6-dimethylenedecahydronaphthalene	C15H24	nd	nd	nd	nd	nd	nd	0.2165
355	2-[1-(Adamantan-1-ylamino)-2,2,2-trifluoro-ethylidene]-malononitrile	C15H16F3N3	nd	nd	nd	nd	nd	nd	0.2053
356	Farnesyl bromide	C15H25Br	nd	nd	nd	nd	nd	nd	0.1983
357	Longiverbenone	C15H22O	nd	nd	nd	nd	nd	nd	0.0950
358	Junenol	C15H26O	nd	nd	nd	nd	nd	nd	0.0625
359	Phenanthrene, 7-ethenyl-1,2,3,4,4a,4b,5,6,7,9,10,10a-dodecahydro-1,1,4a,7-tetramethyl-, [4aS-(4aà,4bá,7á,10aá)]-	C20H32	nd	nd	nd	nd	nd	nd	0.0479
360	Threo-9,10-dihydroxyoctadecanoic acid	C18H36O4	nd	nd	nd	nd	nd	nd	0.0420
361	(1aR,3aS,7S,7aS,7bR)-1,1,3a,7-Tetramethyldecahydro-1H-cyclopropa[a]naphthalen-7-ol	C15H26O	nd	nd	nd	nd	nd	nd	0.0139
362	(R)-lavandulyl acetate	C12H20O2	nd	nd	nd	nd	nd	nd	tr
363	1,7,7-Trimethylbicyclo[2.2.1]hept-5-en-2-one	C10H14O	nd	nd	nd	nd	nd	nd	tr
364	1,1,4,7-Tetramethyldecahydro-1H-cyclopropa[e]azulene-4,7-diol	C15H26O2	nd	nd	nd	nd	nd	nd	tr
365	Fenretinide	C26H33NO2	nd	nd	nd	nd	nd	nd	tr

366	3,9á:14,15-Diepoxypregn-16-en-20-one, 3,11á,18-triacetoxy-	$C_{27}H_{34}O_9$	nd	nd	nd	nd	nd	nd	tr
367	Phenylalanine, 4-amino-N-t-butyloxycarbonyl-, t-butyl ester	$C_{18}H_{28}N_2O_4$	nd	nd	nd	nd	nd	nd	tr
368	Phenol, 3,5-bis(1,1-dimethylethyl)-	$C_{14}H_{22}O$	nd	nd	nd	nd	nd	nd	tr
369	9-Octadecenoic acid, (2-phenyl-1,3-dioxolan-4-yl)methyl ester, trans-	$C_{28}H_{44}O_4$	nd	nd	nd	nd	nd	nd	tr

"nd" indicates that the substance was not detected under analytical conditions used; "tr", trace (<0.01%)

Supplementary Table S2. Relative content (%) of differential metabolites of seven Lamiaceae plant hydrosols.

No.	compound name	Tv HD	Tm HD	Mp HD	Mo HD	Ro HD	Se HD	La HD
1	(-)-Spathulenol	0.01401	0.11765	0.064009	2.205837	0.071774	2.802394	1.341928
2	Caryophyllene oxide	0.277498	0.156312	0.134865	1.264009	0.047988	2.426255	0.668724
3	Thymol	18.84038	19.5366	0	1.367523	0.069203	5.320654	1.091876
4	α-Cadinol	0.03082	0.021817	0.202994	0.623824	0.032565	0.46114	0.578713
5	Terpinen-4-ol	0	4.22033	0	0.552772	0.0001	0.368022	0.180324
6	1-Octen-3-ol	1.613522	1.907123	0.027349	0	0	0	0
7	Benzene, 2-methoxy-4-methyl-1-(1-methylethyl)-	6.276236	2.240502	0	0	0	0	0
8	o-Cymene	2.398435	0.341036	0	0	0	0	0
9	Phenol, 2-methyl-5-(1-methylethyl)-	1.856015	1.868988	0.180678	0	0	0.0001	0
10	2,6-Octadienal, 3,7-dimethyl-, (Z)-	0	0	0	0.679711	0	1.683126	0
11	2-Cyclohexen-1-one, 3-methyl-6-(1-methylethylidene)-	0.0001	0	0.106602	0	0.045177	0	0
12	3-Cyclohexene-1-carboxaldehyde, 1,3,4-trimethyl-	0.150194	0	0	0	0	0.017408	0
13	Benzene, 1-methyl-4-(1-methylethenyl)-	0.059654	0	0	0	0.02374	0	0
14	Butanoic acid, 4-pentenyl ester	0.128713	0.0001	0	0	0	0	0
15	Caryophyllene	0.182264	0	0.130751	0	0	0	0
16	ç-Muurolene	0.116118	0	0.0001	0	0	0	0
17	Cyclohexanol, 5-methyl-2-(1-methylethenyl)-	0.0001	0.011759	0	0	0.0001	0	0
18	Phenol, 5-methyl-2-(1-methylethyl)-, acetate	0.074789	0.402967	0	0	0	0	0
19	exo-2,7,7-trimethylbicyclo[2.2.1]heptan-2-ol	0	0.04446	0	0	0	0.0001	0
20	Phenol, 2-methoxy-3-(2-propenyl)-	0	0.233054	0	0	0	0	0.05372
21	(S)-2,2,6-Trimethyl-6-((S)-4-methylcyclohex-3-en-1-yl)dihydro-2H-pyran-3(4H)-one	0	0	0.0001	0.257715	0.035906	0	0.435725
22	1,4-Methanoazulen-3-ol, decahydro-1,5,5,8a-tetramethyl-, [1S-(1α,3α,3aα,4α,8aα)]-	0	0	0.0001	0	0.0001	0	0

23	1H-2,8a-Methanocyclopenta[a]cyclopropa[e]cyclodecen-11-one, 1a,2,5,5a,6,9,10,10a-octahydro-5,5a,6-trihydroxy-1,4-bis(hydroxymethyl)-1,7,9-trimethyl-, [1S-(1α,1aα,2α,5α,5aα,6α,8aα,9α,10aα)]-	0	0	0.018152	0	0.012501	0	0
24	(1aR,4S,4aR,7R,7aS,7bS)-1,1,4,7-Tetramethyldecahydro-1H-cyclopropa[e]azulen-4-ol	0	0	0	0.346675	0	0	0.531467
25	Cyclohexanol, 2-methyl-5-(1-methylethenyl)-, (1α,2α,5α)-	0	0	0	0.404762	0	0.30067	0
26	Propanoic acid, 2-methyl-, (dodecahydro-6a-hydroxy-9a-methyl-3-methylene-2,9-dioxoazuleno[4,5-b]furan-6-yl)methyl ester, [3aS-(3aα,6α,6aα,9aα,9bα)]-	0	0	0	0.038631	0	0	0.035684
27	Terpineol	0	0	0	0.43236	0	0.339362	0

Supplementary Figures

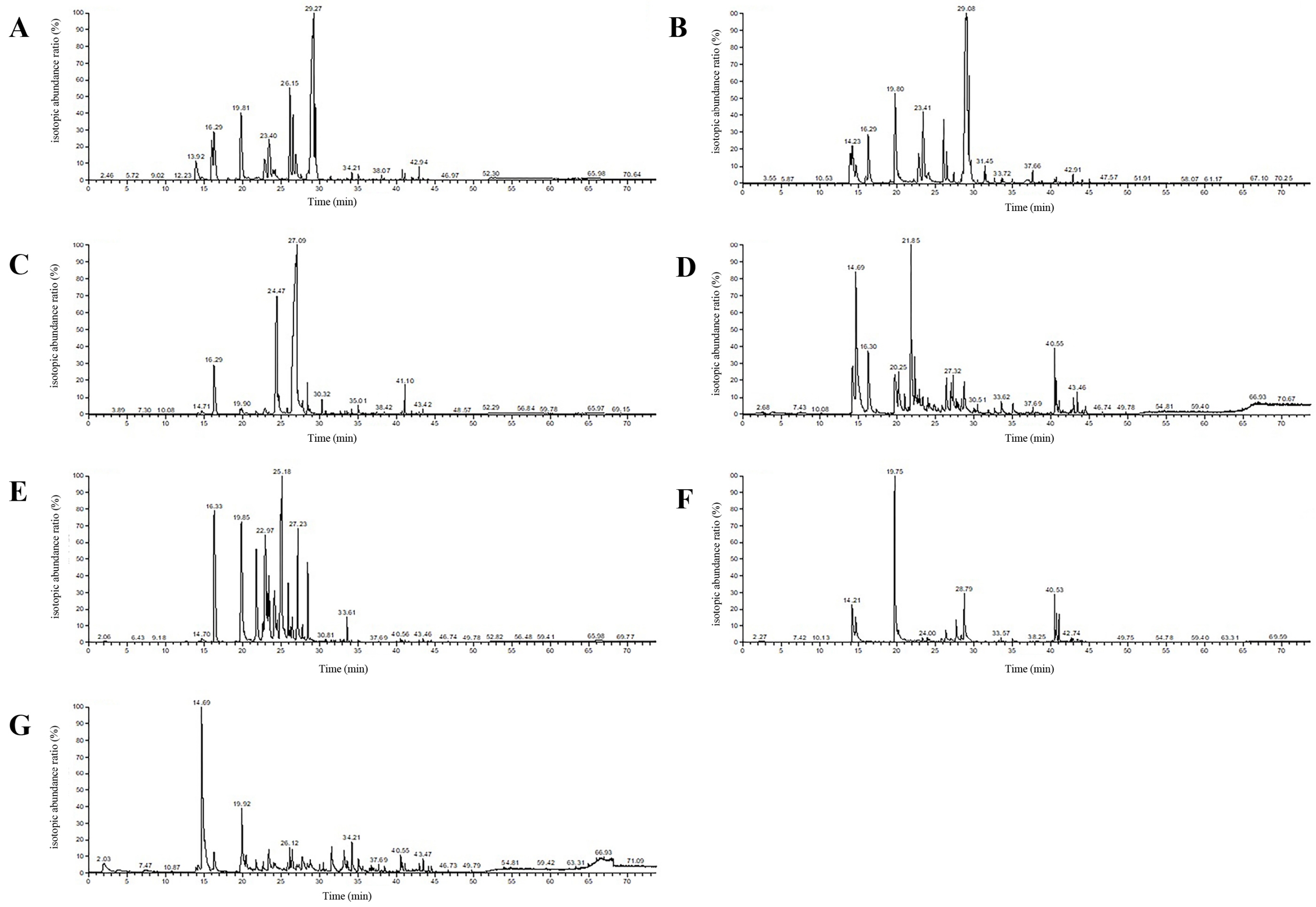

Supplementary Figure S1. Total ion current diagram of seven Lamiaceae plant hydrosols. (A) *Thymus vulgaris*; (B) *Thymus mongolicus*; (C) *Mentha×piperita*; (D) *Melissa officinalis*; (E) *Rosmarinus officinali*; (F) *Salvia elegans*; (G) *Leonurus artemisia*.
